# Efficacy and Safety of a Dexamethasone Implant in Patients with Diabetic Macular Edema at Tertiary Centers in Korea

**DOI:** 10.1155/2016/9810270

**Published:** 2016-05-17

**Authors:** Byung Gil Moon, Joo Yong Lee, Hyeong Gon Yu, Ji Hun Song, Young-Hoon Park, Hyun Woong Kim, Yong-Sok Ji, Woohyok Chang, Joo Eun Lee, Jaeryung Oh, Inyoung Chung

**Affiliations:** ^1^Department of Ophthalmology, Asan Medical Center, University of Ulsan College of Medicine, 88 Olympic-ro 43 gil, Songpa-gu, Seoul 05505, Republic of Korea; ^2^Department of Ophthalmology, Seoul National University Hospital, Seoul National University College of Medicine, 101 Daehak-ro, Jongno-gu, Seoul 03080, Republic of Korea; ^3^Department of Ophthalmology, Ajou University School of Medicine, 164 World Cup-ro, Yeongtong-gu, Suwon 16499, Republic of Korea; ^4^Department of Ophthalmology, Seoul St. Mary's Hospital, The Catholic University of Korea College of Medicine, 222 Banpo-daero, Seocho-gu, Seoul 06591, Republic of Korea; ^5^Department of Ophthalmology, Busan Paik Hospital, Inje University, College of Medicine, 75 Bokji-ro, Busanjin-gu, Busan 47392, Republic of Korea; ^6^Department of Ophthalmology, Chonnam National University Medical School and Hospital, 42 Jebong-ro, Dong-gu, Gwangju 61469, Republic of Korea; ^7^Department of Ophthalmology, Yeungnam University Medical Center, 170 Hyeonchung-ro, Nam-gu, Daegu 42415, Republic of Korea; ^8^Department of Ophthalmology, Haeundae Paik Hospital, Inje University College of Medicine, 875 Haeun-daero, Haeundae-gu, Busan 48108, Republic of Korea; ^9^Department of Ophthalmology, Korea University Anam Hospital, 73 Inchon-ro, Seongbuk-gu, Seoul 02841, Republic of Korea; ^10^Department of Ophthalmology, Gyeongsang National University Hospital, 79 Gangnam-ro, Jinju-si, Gyeongsangnam-do 52727, Republic of Korea

## Abstract

*Purpose*. To evaluate the real-world efficacy and safety of the dexamethasone implant (DEX implant) in patients with diabetic macular edema (DME).* Methods*. Retrospective, multicenter, and noncomparative study of DME patients who were treated with at least one DEX implant. A total of 186 eyes from 165 patients were included. Best-corrected visual acuity (BCVA), central retinal thickness (CRT), complications, and number of retreatments were collected. Data at baseline and monthly for 6 months were analyzed.* Results.* The average baseline BCVA and CRT were 0.60 LogMAR and 491.6 *μ*m, respectively. The mean BCVA improved until 3 months and then decreased up to 6 months of follow-up (0.53, 0.49, and 0.55 LogMAR at 1, 3, and 6 months; *p* = 0.001, <0.001, and 0.044, resp.). The change of mean CRT was similar to BCVA (345.0, 357.7, and 412.5 *μ*m at 1, 3, and 6 months, *p* < 0.001, <0.001, and <0.001, resp.). 91 eyes (48.9%) received additional treatment with anti-VEGF or DEX implant. The average treatment-free interval was 4.4 months. In group analyses, the DEX implant was more effective in pseudophakic eyes, DME with subretinal fluid (SRF), or diffuse type.* Conclusions.* Intravitreal dexamethasone implants are an effective treatment for patients with DME, most notably in pseudophakic eyes, DME with SRF, or diffuse type. A half of these patients require additional treatment within 6 months.

## 1. Introduction

Visual impairment in diabetic retinopathy (DR) is most commonly associated with diabetic macular edema (DME), which affects about 20% of these patients [1]. Recently, anti-VEGF agents have been widely used in the treatment of DME based on the results of clinical trials [2–4]. However, decreasing the VEGF levels alone is not sufficient to reduce DME, and repeated anti-VEGF injections create a burden for patients [5]. Furthermore, longstanding treatment due to the chronic nature of DME can cause resistance to anti-VEGF treatment and tachyphylaxis can develop [6]. Thus, the targeting of inflammatory mediators other than VEGF is becoming a prominent issue for clinicians.

The 0.7 mg dexamethasone (DEX) intravitreal implant is a sustained-release corticosteroid that was developed to achieve longstanding anti-inflammatory effects. Multicenter clinical trials have reported its effectiveness in the treatment of persistent DME [7–10]. However, since these results were derived from a fixed injection regimen and excluded patients with good visual acuity and monthly based follow-ups, we cannot directly apply them to our own clinical setting. Moreover, clinical trials have not evaluated DME with vitrectomized eyes or made comparisons between the types or characteristics of DME.

Hence, we here evaluated the efficacy and safety of the DEX implant in patients with DME in various clinical settings and compared the clinical outcomes according to DME type and clinical situation.

## 2. Materials and Methods

This study was designed as a multicenter, retrospective, noncomparative, and interventional case series. We used data from the eyes of patients diagnosed with DME who were treated with at least one DEX implant at one of 10 tertiary medical centers in Korea. The study adhered to the tenets of the Declaration of Helsinki and was approved by the institutional review board at each center.

The following data were collected: baseline demographic characteristics; past medical and ocular history; treatment history of DME; ophthalmologic examinations including best-corrected visual acuity (BCVA), intraocular pressure (IOP), parameters and characteristics of spectral domain optical coherence tomography (SD-OCT), retreatments, and related complications at baseline and monthly for 6 months. Inclusion criteria were (1) a diagnosis of type 1 or 2 diabetes and (2) center-involved DME treated with one or more intravitreal DEX implants and followed up for at least 6 months. Exclusion criteria were ME (1) secondary to other retinal diseases such as retinal vein occlusion, uveitis, or pseudophakic cystoid ME and (2) accompanied by epiretinal membrane with tractional component or vitreomacular traction syndrome.

Since the 10 tertiary centers used different types of SD-OCT (Spectralis, Cirrus, or 3D-OCT), the central retinal thickness (CRT) was converted in the Spectralis profiles based on a preexisting study [11]. Diffuse ME was defined as two or more disc areas of retinal thickening involving the center of the macula; focal DME was defined as an area of retinal thickening less than two disc areas in diameter affecting the center of the macula [12].

We analyzed the mean changes in the BCVA and CRT from baseline and monthly for 6 months. And the mean change in the IOP, proportion of cases stratified by the extent of the IOP increase, and cataract progression were also evaluated. Cataract progression was defined as an increased severity of lens opacity compared with baseline or previous visit using the descriptive data from the medical charts. Group analyses were performed by comparison of treatment naïve versus nonnaïve eyes, phakic versus pseudophakic eyes, vitrectomized versus nonvitrectomized eyes, the presence versus absence of SRF, and focal versus diffuse type of DME.

Data manipulation and statistical analyses were conducted using SAS® version 9.3 (SAS Institute Inc., Cary, NC). To estimate the effects of both time and group on the outcomes, we employed the linear mixed model that accounted for patient effects. If the group-by-time interaction effect was significant, the time effects within groups and group effects within time points were compared. If the group-by-time interaction effect was not significant, it was excluded from the analyses. All of the reported *p* values were two sided, and *p* values less than 0.05 were considered to indicate statistical significance.

## 3. Results

**Figure 1:**
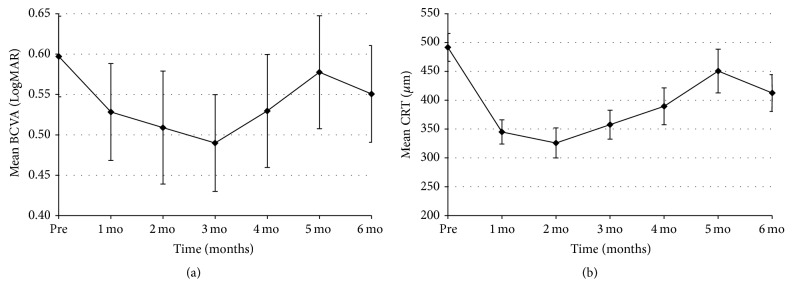
Mean changes in the LogMAR BCVA and CRT in the study eyes.

**Figure 2:**
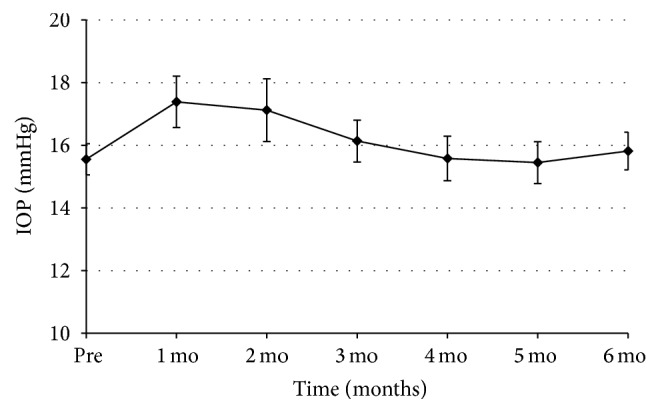
Mean changes in the IOP in the study eyes.

A total of 210 eyes from 189 patients with DME were collected from 10 tertiary medical centers. Of these, 186 eyes (from 165 patients) satisfied the inclusion criteria. Detailed baseline characteristics of the study eyes are provided in Table 1. The mean patient age was 57.8 ± 11.5 years, and the mean duration of DME was 17.3 ± 22.8 months. The average baseline BCVA and CRT values were 0.60 ± 0.36 LogMAR and 491.6 ± 164.6 *μ*m, respectively. Compared to the value at baseline, the mean BCVA gradually improved for 3 months and then decreased up to the 6-month follow-up (0.53 ± 0.39, 0.49 ± 0.37, and 0.55 ± 0.38 LogMAR at 1, 3, and 6 months; *p* = 0.001, <0.001, and 0.044, resp.; Figure 1). And the mean CRT significantly decreased from baseline after injection throughout the entire follow-up period (345.0 ± 125.1, 357.7 ± 137.7, and 412.5 ± 180.8 *μ*m at 1, 3, and 6 months; *p* < 0.001, <0.001, and <0.001, resp.; Figure 1). The thinnest average CRT was present at 2 months (325 ± 119.2 *μ*m) and gradually thickened from 3 months. The mean baseline IOP was 15.6 ± 3.4 mmHg. Compared with baseline, the mean IOP was higher at the 1-month and 2-month follow-up visits (17.4 ± 5.3 and 17.1 ± 5.0 mmHg; *p* < 0.001 and <0.001, resp.), but after 2 months, there was no statistically significant difference in the mean IOP (Figure 2).

### 3.1. Adverse Events

Eight eyes (4.3%) had an IIOP of more than 30 mmHg, and one eye had an IOP that increased to 50 mmHg at 1 month after the DEX implantation. This patient was managed using anterior chamber paracentesis with IOP lowering agents and then maintained a normal IOP range. All of the other patients were managed with one or two IOP lowering agents. In the 112 phakic eyes, 26 (23.2%) eyes showed progression of lens opacity and 7 (6.3%) received lens extraction during the study period. Infectious endophthalmitis occurred in one patient (0.5%) in the study eye 3 days after the DEX implantation and was subsequently managed with implant removal combined with vitrectomy and injection of intravitreal antibiotics. No other complications such as vitreous hemorrhage, retinal tear, or retinal detachment were reported.

### 3.2. Retreatment during the Study Period

After the first injection of the DEX implant, 91 eyes (48.9%) received additional anti-VEGF treatment or a DEX implant. The average treatment-free interval was 4.4 ± 1.5 (range, 1–6) months. The mean number of total retreatments with anti-VEGF drugs or a DEX implant was 0.68 (range 0–4) during the 6-month study period. There was a mean of 0.42 (range, 0–4) anti-VEGF injections for 51 eyes (27.4%) and 0.27 (range, 0-1) DEX implant reinjections for 49 eyes (26.3%). Six eyes (3.2%) were retreated with both anti-VEGF and DEX implants because of persistent DME.

### 3.3. Group Analysis

**Figure 3:**
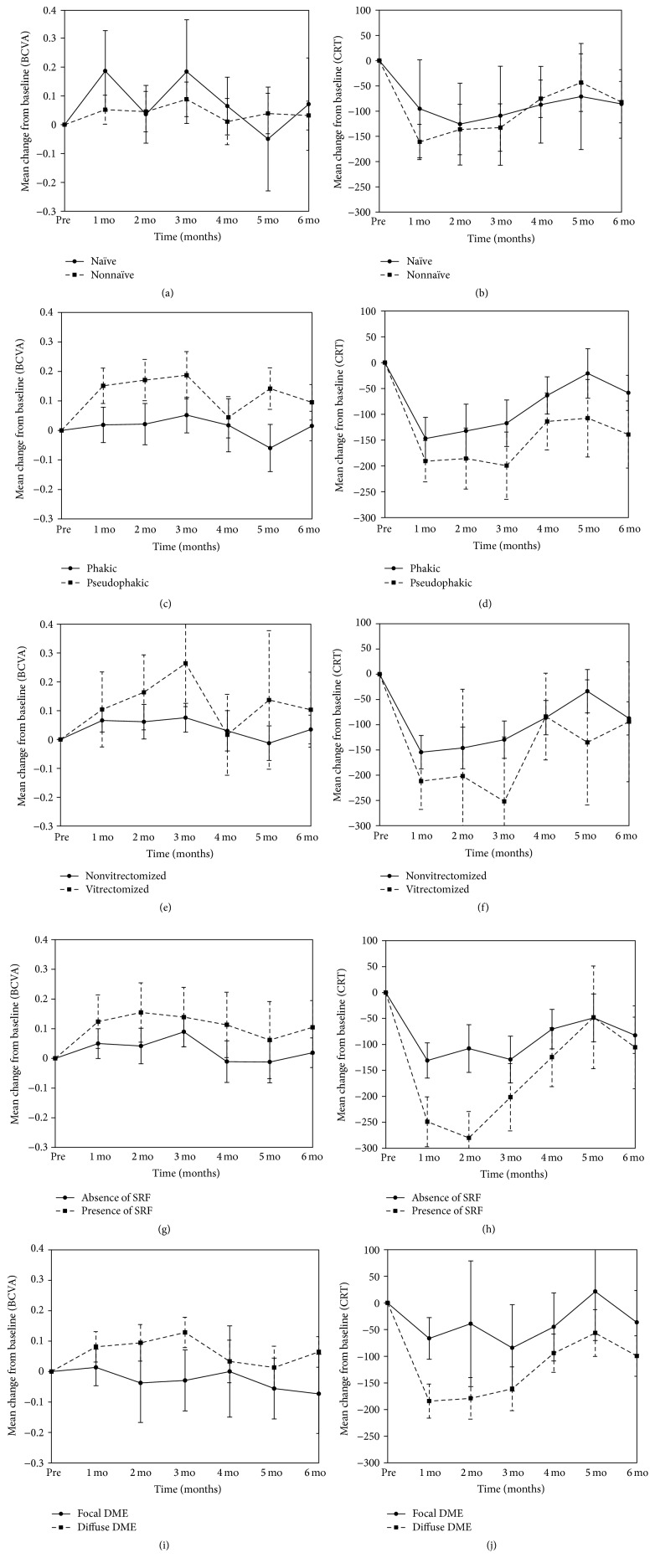
Mean changes from baseline BCVA and CRT in various group analyses. (a) and (b) Treatment naïve versus nonnaïve eyes; (c) and (d) phakic versus pseudophakic eyes; (e) and (f) nonvitrectomized versus vitrectomized eyes; (g) and (h) absence of SRF versus presence of SRF; (i) and (j) focal versus diffuse DME.

**Figure 4:**
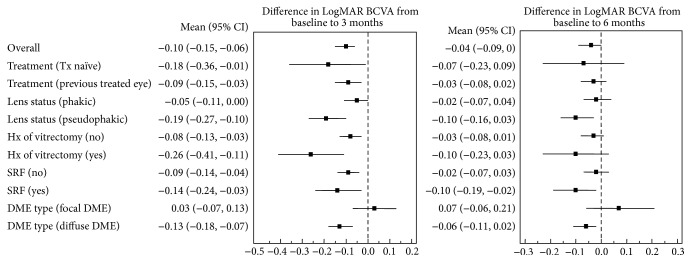
Forest plots of the mean change in BCVA (95% confidential interval) from baseline to months 3 and 6.

**Figure 5:**
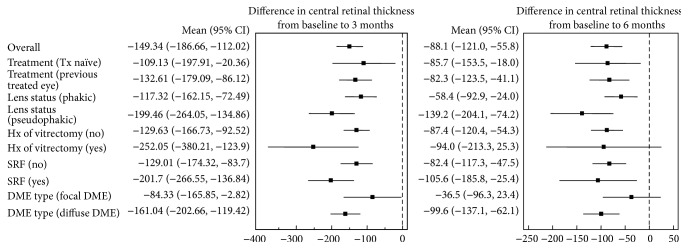
Forest plots of the mean change in CRT (95% confidential interval) from baseline to months 3 and 6.

Five sets of group analyses were performed: naïve versus nonnaïve eyes, pseudophakic versus phakic eyes, vitrectomized versus nonvitrectomized eyes, the presence versus the absence of SRF, and focal versus diffuse DME. The mean changes in BCVA from baseline in these five groups are shown in Figures 3 and 4 (functional results), and the mean changes in CRT from baseline are shown in Figures 3 and 5 (anatomical results).

### 3.4. Functional Results of the Group Analyses

Thirty-one eyes (16.7%) did not undergo prior treatment for DME. The mean change in LogMAR BCVA from baseline was not significantly different during follow-up between naïve and nonnaïve eyes (*p* = 0.609, Figure 3(a)). Seventy-four eyes (39.8%) were pseudophakia at baseline examination. Compared with the phakic eyes, the mean change in LogMAR BCVA did not differ over time (*p* = 0.657); however, the overall difference in LogMAR BCVA from baseline was greater in the pseudophakic eyes (mean difference = 0.113; *p* = 0.001, Figure 3(c)). Thirty-one study eyes (16.7%) had a history of previous vitrectomy for proliferative diabetic retinopathy (PDR). The mean change in the LogMAR BCVA did not significantly differ between vitrectomized and nonvitrectomized eyes over time or in terms of an overall change (*p* = 0.472 and 0.210, resp., Figure 3(e)). A total of 56 eyes (30.1%) had the SRF type of DME at the baseline examination. The group with SRF at baseline showed significantly improved changes in overall mean LogMAR BCVA values compared with the group lacking SRF (mean difference = 0.086; *p* = 0.019, Figure 3(g)). Twenty-seven eyes (14.5%) had focal DME at the baseline examination. Over time, the mean change in LogMAR BCVA from baseline was not significantly different between focal and diffuse DME eyes (*p* = 0.247). However, the overall difference in the mean BCVA change was greater in the diffuse DME group than in the focal DME group (mean difference = 0.107; *p* = 0.025, Figure 3(i)).

### 3.5. Anatomical Results of Group Analyses

The mean overall change in CRT was not different in our analyses of treatment naïve versus nonnaïve eyes (*p* = 0.475, Figure 3(b)), pseudophakic versus phakic eyes (*p* = 0.088, Figure 3(d)), and vitrectomized versus nonvitrectomized eyes (*p* = 0.157, Figure 3(f)) throughout the follow-up period. When the influence of SRF was evaluated, the mean decrease in CRT was significantly different between the two groups over time (*p* = 0.005). Therefore, we compared the mean change in the CRT at different time points and found that the mean decrease in the CRT was greater in the (+) SRF group after 1, 2, and 3 months (mean difference = 121.9, 139.8, and 96.7 *μ*m, resp.; *p* < 0.001, <0.001, and 0.006, resp., Figure 3(h)). Finally, there were no significant differences in the CRT changes between the focal and diffuse DME eyes over time (*p* = 0.358). However, the mean overall decrease in CRT was greater in the diffuse DME group than in the focal DME group (mean difference = 85.5 *μ*m; *p* = 0.003, Figure 3(j)).

### 3.6. Group Analyses of Retreatment Rates

Retreatment with anti-VEGF agents or a repeated DEX implant over the 6-month study period was compared between the groups and found not to significantly differ between naïve versus nonnaïve, nonvitrectomized versus vitrectomized, or phakic versus pseudophakic eyes. However, the retreatment rates were higher in (+) baseline SRF groups and the diffuse DME than in the (−) baseline SRF groups and the focal DME (*p* = 0.020 and *p* < 0.001, resp.; Table 2).

## 4. Discussion

In our current clinic-based study, the DEX implant was found to be effective for treating DME with regard to visual gain and decreasing ME. Visual outcomes with this treatment were more effective in pseudophakic eyes and eyes with a baseline SRF or diffuse DME, and anatomical outcomes were better in eyes with baseline SRF or diffuse DME.

The DEX implant is widely used as an alternative to, or in combination with, anti-VEGF treatment. Recent clinical trials have reported promising efficacy and safety of the DEX implant, especially in pseudophakic eyes [9, 10]. However, in clinical practice, some patients cannot be followed up monthly; moreover, the reinjection time and regimens differ by individual and according to the treatment response. Therefore, the results from clinical trials should be carefully applied in clinical field and with detailed consideration of the individual patient. Of note also, most clinical trials did not confirm the influences of a previous vitrectomy, the type of DME, or the presence of baseline SRF.

In our current study therefore, we included pseudophakic or vitrectomized eyes and previously treated eyes to evaluate the efficacy of the DEX implant in treating DME in a real-world setting. The mean duration of DME in our series was about 17 months, which reflects the fact that many cases of recalcitrant DME were included in our study cohort. Among the treated eyes, the mean BCVA improved in the first month with maximal improvement at 3 months, after which it gradually declined during months 4 through 6. This trend in visual acuity is in accordance with preexisting studies on DME [7, 13]. At the 6-month follow-up visit in our present study patients, the mean BCVA became significantly different from baseline since most retreatments were performed before 6 months due to recurrence of DME. The mean CRT showed a similar and mirroring trend to the changes in BCVA. The thinnest CRT was seen at the 2-month follow-up visit and then gradually increased through 6 months. These results confirmed the fact that anatomical improvement preceded functional improvement.

An increased IOP as a result of a DEX implant has been reported to be transient and manageable in most studies [14–16]. Our current study findings showed that the mean IOP was higher after 1 and 2 months compared to baseline in our DME cases. About 4% of our current study eyes showed an IOP greater than 30 mmHg, and only one eye had an IOP increase of up to 50 mmHg 1 month after injection. This patient was well managed using an anterior chamber tap and IOP lowering agents, with other patients being managed with one or two IOP lowering agents. None of the eyes underwent filtering surgery during the study period.

The development and progression of cataract are secondary side effect of the DEX implant. Our current study showed that 26 eyes (23%) had lens opacity progression from baseline, and 7 eyes (6%) required cataract extraction surgery within 6 months. Although our current study had only a short-term follow-up, the incidence of cataract progression was relatively higher than reported in previous retrospective studies [13, 14, 16, 17]. We could not know the exact grade of the lens opacity at each visit due to the retrospective and multicenter nature of the data. Since the proportion of cataract surgeries was similar in our current series to that of a previous report, overall incidence of cataract progression could be possible to overestimate by some inappropriate data [17]. Long-term follow-ups and more exact grading studies are needed to precisely identify the incidence of cataract progression in DME patients in clinical settings.

Due to the multicenter and retrospective design of our current analysis, retreatment and the injection regimen were selected at the discretion of the treating physician. Prior clinical trials have used at least a 6-month fixed interval for DEX implant reinjection [9, 10]. However, since DME has a chronic and multifactorial nature, most studies have reported an optimal reinjection interval of less than 5 months [9, 18]. Our present study showed similar results in that the retreatment interval was 4.4 months, and almost half of the eyes (49%) required retreatment within 6 months. Therefore, it is not necessary for physicians to wait 6 months to retreat patients. In fact, many studies recommend reinjection prior to 5 months because delayed retreatment can cause insufficient functional and anatomical gains [9, 16, 18].

Improvement in visual acuity in patients treated with the DEX implant is more prominent in a pseudophakic eye as there is no effect on the lens. In our current study, there was significant improvement in the BCVA in pseudophakic eyes compared to the phakic group. We concluded that the DEX implant is the more reliable treatment choice for pseudophakic DME.

The efficacy of the DEX implant does not differ between vitrectomized and nonvitrectomized eyes, based on the findings of previous reports [19, 20]. In our present study, improvement in the BCVA and decreases in CRT were not different between these two groups. Therefore, the DEX implant appears to be a good treatment option for DME in patients who have previously undergone a vitrectomy.

The presence of SRF is a common finding in diffuse DME cases and responds well to anti-VEGF or corticosteroid treatment. The mean improvement in the BCVA and CRT was significantly better in the group with SRF in our current study. Baseline SRF might contribute to poor initial visual acuity and thick CRT, but also this group showed a better response to the DEX implant. Hence, patients who have the SRF type of DME are more suitable candidates for a DEX implant as they are likely to show a good response to this treatment. The results of a previous phase II study also showed that the DEX implant had a similar efficacy in terms of focal and diffuse DME [21]. However, we here observed better functional and anatomical outcomes in diffuse DME than focal DME. Therefore, this subtype of DME is suitable for the DEX implantation.

Our study had several limitations of note. First, due to the retrospective nature of our analyses, there may have been a patient selection bias, and the information we were able to collect was limited. Second, due to the collection of data from multiple tertiary centers, a consentaneous follow-up protocol and retreatment criteria were lacking. Third, anti-VEGF medications and DEX implants were used in the retreatment regimens in our current series but our results did not reflect the sole efficacy of the DEX implant. The strength of our current study was its relatively large sample size, which resulted in an acceptable statistical strength. In addition we performed various group analyses in clinical settings, thereby reflecting real-world situations.

## 5. Conclusion

The 0.7 mg dexamethasone implant appears to be beneficial for the treatment of DME with regard to functional and anatomical improvements in clinic-based settings. Although this implant is more effective in pseudophakic eyes, and in eyes with the presence of SRF and the diffuse type of DME, it may also be a possible treatment option for DME in vitrectomized eyes. IIOP in these cases is infrequent and transient and can be controlled by IOP lowering agents.

## Figures and Tables

**Table 1:** Patient demographics and clinical characteristics of the study eyes.

Characteristics	186 eyes (*n* = 165)
Age (range)	57.8 ± 11.5 (25–85)
Male : female (% male)	88 : 98 (47.3%)
Type of DM	
Type 1 DM	14 (7.5%)
Type 2 DM	172 (92.5%)
Mean duration of DM (years)	13.2 ± 8.9
HbA1c (%)	7.1 ± 2.4
Grade of DR	
NPDR (%)	103 (55.4%)
PDR (%)	83 (44.6%)
Mean duration of DME (months)	17.3 ± 22.8
Type of DME	
Focal DME (%)	27 (14.5%)
Diffuse DME (%)	159 (85.5%)
History of vitrectomy (% yes)	31 (16.7%)
History of cataract extraction (% yes)	74 (39.8%)
Mean LogMAR BCVA	0.60 ± 0.36
Mean IOP (mmHg)	15.6 ± 3.4
Mean CRT (*μ*m)	491.6 ± 164.6
Presence of SRF (%)	56 (30.1%)
Previous treatment for DME	
Treatment of naïve patients	31 (16.7%)
Macular laser treatment	29 (15.6%)
Anti-VEGF	152 (81.7%)
Intravitreal steroid	42 (22.6%)

DM, diabetes mellitus; HbA1c, glycated hemoglobin; DR, diabetic retinopathy; NPDR, nonproliferative diabetic retinopathy; PDR, proliferative diabetic retinopathy; DME, diabetic macular edema; LogMAR, logarithm of the minimum angle of resolution; BCVA, best-corrected visual acuity; IOP, intraocular pressure; CRT, central retinal thickness; SRF, subretinal fluid; VEGF, vascular endothelial growth factor.

**Table 2:** Comparison of the retreatment rates by group analysis.

Group analysis	Presence of retreatment (%)	*p* value^*∗*^
Treatment naïve versus nonnaïve	11/31 (35.5%) versus 81/155 (52.3%)	0.088
Pseudophakic versus phakic	37/74 (50.0%) versus 55/112 (49.1%)	0.905
Vitrectomized versus nonvitrectomized	14/31 (45.2%) versus 78/155 (50.3%)	0.600
Presence versus absence of SRF	35/56 (62.5%) versus 57/130 (43.8%)	0.020
Diffuse versus focal DME	88/159 (55.3%) versus 4/27 (14.8%)	<0.001

SRF, subretinal fluid; DME, diabetic macular edema.

^*∗*^Chi square test.

## Additional Points

The intravitreal dexamethasone implant is an effective treatment for diabetic macular edema and yields beneficial anatomical and functional outcomes in clinical settings.
